# Technical Advancement of Radiation Therapy

**DOI:** 10.1155/2014/797412

**Published:** 2014-02-11

**Authors:** Tsair-Fwu Lee, Jack Yang, Eng-Yen Huang, Chung-Chi Lee, Maria F. Chan, An Liu

**Affiliations:** ^1^Medical Physics and Informatics Laboratory, Department of Electronics Engineering, National Kaohsiung University of Applied Sciences, Kaohsiung 807, Taiwan; ^2^Department of Radiation Oncology, Institute for Advanced Radiation Oncology, Monmouth Medical Center, Barnabas Health, Long Branch, NJ 07740, USA; ^3^Department of Radiation Oncology, Kaohsiung Chang Gung Memorial Hospital, Chang Gung University, College of Medicine, Kaohsiung 833, Taiwan; ^4^Department of Medical Imaging and Radiological Sciences, Chang Gung University, Taoyuan 333, Taiwan; ^5^Medical Physics Department, Memorial Sloan-Kettering Cancer Center, 136 Mountain View Boulevard, Basking Ridge, NJ 07920, USA; ^6^Department of Radiation Oncology, City of Hope National Medical Center, Duarte, CA 91010, USA

Radiation therapy is an integrated part of the modern comprehensive cancer management. It has been proven that treatment effectiveness leads to curing various types of cancers. For example, radiotherapy can result in a 2-year progression-free (PF) survival and overall survival rate to 72.7% and 80.2%, respectively, using IMRT techniques for dose painting of nasopharyngeal carcinoma [1]; this is a very sensitive type of tumor to DNA killing by radiation beams. However, the cure rate for very advanced tumors and certain cancers such as glioblastoma multiforme is still pessimistic. Currently, strategies to improve efficacy of radiation therapy are being studied. One of the major improvements of radiation therapy is to increase the treatment dose to the tumor. However, the main limiting factor to higher dosage delivery to tumor cells is normal tissue tolerances. To overcome this limitation, one can reduce treatment volume to normal tissue or sculpt the target dose as an increment with dose to the tumor [2]. This can be achieved by improving the definition of target volume and the precision of dosimetry delivery with advanced treatment techniques.

One of the major advancements in radiation oncology in early 1990s was the development of conformal three-dimensional (3D) radiation therapy. In this technique, the prescribed dose volume was made to conform to the target volume and spare more normal tissues. Furthermore, radiation dosimetry based on 3D conformal therapy has been studied more accurately and Monte Carlo methodology [3] has been also introduced into the current calculation for patient dosimetry. In the last decade, much interest has been generated in other forms of conformal treatment planning, that is, intensity modulated radiation therapy (IMRT) and stereotactic radiosurgery and radiotherapy (SRS and SRT). IMRT involves the delivery of optimized, nonuniform irradiation beam intensities. A uniform dose distribution can be created around the tumor by either modulating the intensity of the beam during its journey through the linear accelerator or by using multileaf collimators. The resultant dose distributions are highly conformal. This technology provides the potential of improved tumor irradiation and sparing of the organ in the vicinity of the target to an extent that was not possible before, especially for concave target volume usually seen in head and neck cancers. In head and neck cancers, tumor dose is often limited by the surrounding critical structures such as spinal cord. Tight dose gradients around the target volume also enable higher doses to be delivered to the tumor while reducing the dose to surrounding critical organs and radiosensitive tissues such as salivary glands, ears, optic chiasm, and hippocampi. Lee et al. [4] in early days reported a locoregional recurrence-free survival of 97% at a median followup of 31 months for 67 nasopharyngeal carcinoma patients treated with IMRT. At 24-month followup, 92% patients had grade 0-1 xerostomia. Other late toxicities were not assessed for the follow-up period. This has indicated that with newer technology and with optimal dose distribution, impressive clinical outcome could be achieved.

While radiotherapy treatment planning technique is matured to a level with precise dosimetry distribution, however, the inaccuracy of CT definition of tumors and normal structures could likely hinder the accurate target delineation and dose calculations. To overcome those limitations, fusion with other imaging techniques, for example, MRI and PET, is being implemented. MRI has been shown to have better differentiation between normal tissues and many different types of tumors. Image fusion studies with FDG-PET and CT scan have shown encouraging results. A significant impact of PET-derived contours on treatment planning has been shown in 30–60% of the plans with respect to the CT-only target volume [5].

SRS and SRT are forms of the 3D technique that deliver radiation doses in one fraction or hypofractionated scheme to a small intracranial target or targets close to critical structure such as spinal cord or brain stem. Both SRS and SRT combine the contemporary principles of neurosurgery and radiotherapy. Patient's head is attached securely to a fixation device. Radiotherapy can be delivered with 3 methods, namely, heavy charged particles (i.e., protons), gamma irradiation emitted from Co-60, and high energy photon irradiation produced with linear accelerators. Conformal radiotherapy is achieved by the use of multiple noncoplanar arcs. SRS and SRT have been proven to be effective in the treatment for controlling tumor growth or even the benign intracranial and extra-cranial diseases.

With the improvement of treatment imaging modalities, to adjust patient localization in a nearly real-time basis becomes feasible. The on-treatment cone/fan beam CT, with patient immobilized at the treatment position, while repositioning correlation based on correlation to the planning CT has become a standard image guided radiation therapy (IGRT) in modern centers. This process has been proven to avoid any positional discrepancy while performing daily patient treatment. Furthermore, with the improvement of CPU/GPU power of current computing technology, the real-time calculation has been making good process. Li [6] has introduced an extensive summary publication on how to approach these adaptive radiation treatment techniques.

There are many topics in the radiation therapy which could be addressed in more details. Also there have been various treatment devices and methodologies introduced to clinical treatment with efficacy, hopefully, reaching better clinical outcomes. The main focus of this special issue is focusing on the new development in cancer treatment, quality control, treatment techniques, and radiation dosimetry with related topics. The special issue covers the most recent developments and ideas in radiation oncology in a broad spectrum, with special emphasis given to the clinical progress with new technologies. This special issue included various topics which have been discussed by researchers as follows:

- clinical trials and outcome research,
- new technologies development and implementation,
- treatment delivery techniques,
- disease specific treatment discussion,
- radiation dosimetry analysis,
- radiation protection, shielding and design,
- clinical therapy physics review and applications,
- molecular imaging application in Radiation Therapy,
- medical imaging,
- professional issues in medical, clinical and biomedical physics,
- radiobiology,
- quality control and assurance,
- computing algorithm and optimization,
- quality of life analysis,
- radiation safety.

This editorial provides comprehensive introduction to these new technology advancements in radiation therapy and many important topics associated with implementing the technologies. In conclusion, the technological advancements in radiotherapy in the last two decades have been immense. There are tremendous amounts of information available via modern technological implementation. Clinical data on these new technologies are proven by many institutions. Again, there will be more technical breakthroughs on cell or even at the molecular levels, which may present another challenge for researchers.

*Tsair-Fwu Lee* *Jack Yang* *Eng-Yen Huang* *Chung-Chi Lee* *Maria F. Chan* *An Liu*
